# Microsurgical Management of Posterior Circulation Aneurysms: A Retrospective Study on Epidemiology, Outcomes, and Surgical Approaches

**DOI:** 10.3390/brainsci12081066

**Published:** 2022-08-11

**Authors:** Wanchun You, Jiahao Meng, Xingyu Yang, Jie Zhang, Guannan Jiang, Zeya Yan, Feng Gu, Xinyu Tao, Zhouqing Chen, Zhong Wang, Gang Chen

**Affiliations:** Department of Neurosurgery & Brain and Nerve Research Laboratory, The First Affiliated Hospital of Soochow University, Suzhou 215006, China

**Keywords:** microsurgical occlusion, posterior circulation aneurysm, surgical approach, lateral supraorbital approach, outcome

## Abstract

Posterior circulation aneurysms have been regarded as the most challenging for endovascular coiling and microsurgical occlusion. The role of microsurgical treatment is gradually being overlooked and diminishing in the trend of endovascular treatment. As microsurgical occlusion of posterior circulation aneurysms is decreasing, we present our relevant experience to evaluate treatment options and surgical approaches. A retrospective study was conducted in the Department of Neurosurgery of the First Affiliated Hospital of Soochow University between 2016 and 2021. Patients with posterior circulation aneurysms treated by clipping, bypass, and trapping were enrolled and followed up for at least six months. We included 50 patients carrying 53 posterior circulation aneurysms, 43 of whom had aneurysm ruptures. The posterior cerebral artery and posterior inferior cerebellar artery were the most common aneurysm locations. Direct clipping was performed in 43 patients, while bypass and trapping was performed in six patients. The retrosigmoid, far-lateral, and midline or paramedian suboccipital approaches were performed for those aneurysms in the middle and lower thirds. Aneurysms in the upper third required the lateral supraorbital approach, pterional approach, subtemporal approach, and occipital craniotomy. The lateral supraorbital approach was utilized in seven patients for aneurysms above the posterior clinoid process. Thirty-four patients recovered well with modified Rankin score 0–3 at discharge. No patient experienced aneurysm recurrence during the mean follow-up period of 3.57 years. Microsurgery clipping and bypass should be considered in conjunction with endovascular treatment as a treatment option in posterior circulation aneurysms. The lateral supraorbital approach is a feasible, safe, and simple surgical approach for aneurysms above the posterior clinoid process.

## 1. Introduction

Subarachnoid hemorrhage (SAH) is a common disease of the central nervous system, which accounts for about 5–10% of all stroke patients [1], and the population-wide incidence is approximately 1–2% [2]. The age of the main population suffering from SAH is significantly younger than those who experience other types of stroke [3]. About one-third of patients with SAH have a reduced quality of life [4]. Ruptured intracranial aneurysm is the leading cause of non-traumatic SAH [5]. Although posterior circulation aneurysms account for, at most, 15% of all intracranial aneurysms [2], posterior circulation aneurysms have a higher risk of rupture and a worse clinical outcome than anterior circulation aneurysms [6,7,8,9]. The common sites of posterior circulation aneurysm are, in order, basilar artery (BA), posterior cerebral artery (PCA), superior cerebellar artery (SCA), and posterior inferior cerebellar artery (PICA). In contrast, aneurysms of the vertebral artery (VA) and anterior inferior cerebellar artery (AICA) are relatively rare.

With advances in endovascular techniques and increased clinician experience for intracranial aneurysms, the rate of endovascular coiling has been gradually increasing [10]. Based on the International Subarachnoid Aneurysm Trial (ISAT), endovascular coiling is currently seen as a better therapeutic option to achieve survival free of disability [8,11,12]. However, the microsurgical treatment appears superior for lower rates of rebleeding, lower rates of recurrence, and higher rates of aneurysm obliteration [8,11,12,13]. It is worth noting that posterior circulation aneurysms remain a formidable challenge for microsurgical treatment because of their hidden and deep location, difficulty in anatomical exposure, the narrow field for microsurgical operation, and the close relationship with the brain stem and hypothalamus. Hence, due to the rarity of microsurgical occlusion for posterior circulation aneurysms, we summarized 50 patients with posterior circulation aneurysms who underwent microsurgical treatment at our hospital between 2016 and 2021 to assess this therapeutic option and corresponding surgical approaches. 

## 2. Materials and Methods

### 2.1. Study Design

This study was conducted at the First Affiliated Hospital of Soochow University, an academic tertiary care hospital specializing in cerebrovascular diseases, and obtained approval from the Medical Ethics Committee. For the retrospective type of study and non-reporting of patient-identifiable data, consent of patients was not required. We performed the retrospective review through a prospectively collected and maintained electronic medical record. All patients received microsurgical treatment for at least one posterior circulation aneurysm between January 2016 and December 2021. Our study included all saccular, fusiform, and dissecting aneurysms located in BA, PCA, SCA, AICA, PICA, and VA. The usage of digital subtraction angiography (DSA), computed tomography angiography (CTA), or magnetic resonance angiography (MRA) confirmed the presence, number, size, morphology, and location of aneurysms. In patients with a suspected ruptured aneurysm, subarachnoid, cerebral cistern, or intraventricular hemorrhage was confirmed by a computed tomography (CT) scan. CT and magnetic resonance imaging (MRI) were capable of identifying thrombotic aneurysms. The patients with traumatic aneurysms, mycotic aneurysms, or any other diagnosed cerebrovascular disease were excluded. The study also excluded patients with incomplete clinical or imaging information. 

### 2.2. Data Extraction

Two independent authors separately extracted the data from the electronic medical record and radiographic database. Prospectively collected and retrospectively analyzed demographic, clinical, or aneurysm characteristics included the number of patients, age, sex ratio, clinical presentation, comorbidities, Hunt and Hess grade at presentation, modified Fisher grade at presentation, preoperative modified Rankin score (mRS), total number of aneurysms, number of patients with multiple aneurysms, aneurysm morphology, size of the saccular aneurysm, aneurysm location, aneurysm side, number of thrombotic aneurysms, surgical approach, treatment technique, usage of intraoperative adjunct, duration of surgery, intra-procedural rupture, postoperative complications, aneurysm obliteration, and re-operation. 

Clinical presentation included headache, nausea and vomiting, alteration of consciousness, hoarseness and dysphagia, limb weakness, and seizure. The severity of SAH was evaluated using the Hunt and Hess grade [14], and the modified Fisher grade [15] was used to assess the appearance of SAH and intraventricular hemorrhage on the CT scan at presentation. Aneurysm morphology was classified into the saccular aneurysm, fusiform aneurysm, and dissecting aneurysm. Size of the saccular aneurysm, aneurysm location, aneurysm side, thrombotic aneurysm, and aneurysm obliteration were confirmed by at least one neurosurgeon and one neuroradiologist from radiographic data. Both indocyanine green videoangiography and microvascular Doppler were alternative intraoperative adjuncts. Since 2019, we have used the FLOW 800 integrated into a surgical microscope (Pentero 900, Carl Zeiss Co., Oberkochen, Germany) to analyze cerebral blood flow based on indocyanine green videoangiography. Postoperative complications we collected included rebleeding, new neurological deficit, hydrocephalus, intracranial infection, and cerebrospinal fluid leak. 

### 2.3. Outcome and Follow-Up

The mRS was used to evaluate functional recovery at discharge. mRS 0–3 represented good clinical outcomes and functional independence, while mRS 4–5 represented poor clinical outcomes [15]. mRS 6 meant death. Apart from patients lost to follow-up, the remaining patients were followed up at least once at six months by a direct interview in outpatient or telephone contact. The angiography at the last follow-up confirmed aneurysm recurrence. Length of follow-up was defined as the time from surgery to the last follow-up.

### 2.4. Surgery and Approach

Except for patients who had suffered life-threatening brain herniation and were in urgent need of surgery, the preferred surgical option was derived through departmental discussions, based on demographic, clinical, and aneurysm characteristics. Neurosurgeons specializing in endovascular embolization and microsurgical occlusion would provide recommendations and reach a consensus agreement. The ultimate surgery plan depended on the preferences of the patient and family. In general, we tended toward microsurgery treatment for ruptured aneurysms, multiple aneurysms, more giant aneurysms, thrombotic aneurysms, wide-neck aneurysms, and patients with neurological deficit or local mass effect. Among this group of patients, clipping was the highest priority. In patients with fusiform aneurysms, dissecting aneurysms, and thrombotic aneurysms that were hard to clip, we preferred bypass and trapping if microsurgical clipping was not feasible. The surgeons chose low-flow or high-flow bypass depending on the blood flow. Intraoperative indocyanine green videoangiography and microvascular Doppler were performed to confirm the patency of the anastomotic site, parent artery, and bypass. Trapping and thrombectomy were appropriate for fusiform or thrombotic aneurysms located at the artery’s end.

For surgical approaches, aneurysm location, size of the aneurysm, critical neural structures nearby, the direction of the aneurysm apex, and preferences of the treating neurosurgeon were considered. Despite the various surgical approaches available, it was equally crucial to consider sharp dissection of the arachnoid, meticulous exposure of the aneurysm neck, maximal preservation of the parent, branching, and perforating artery patency, minimal neural injury, and complete aneurysm obliteration.

## 3. Results

### 3.1. Demographic and Clinical Characteristics

From 1 January 2016 to 31 December 2021, a total of 50 patients with posterior circulation aneurysms were included in this retrospective study. The mean age for each patient was 54.92 ± 10.92 (range: 25 to 77 years). A female preponderance was observed (*n* = 28, 56%). The most common presentation was headache (*n* = 40, 80%). Thirty-one patients (62%) presented with nausea and vomiting. Twenty-two patients (44%) were admitted to our hospital with alteration of consciousness, while patients who presented with hoarseness and dysphagia (*n* = 1, 2%), limb weakness (*n* = 2, 4%), and seizure (*n* = 1, 2%) were relatively few. Hypertension (*n* = 20, 40%) was frequently seen as a comorbidity. Regarding clinical characteristics, 44% of patients at admission were in poor clinical grade (Hunt and Hess grade III, IV, and V), and the median was grade III. The median admission modified Fisher grade was 3 (interquartile range [IQR] 1–4). All the demographic and clinical characteristics are available in Table 1. 

### 3.2. Aneurysm Characteristics

Out of 53 posterior circulation aneurysms, there were 43 ruptured aneurysms (81.13%), while unruptured aneurysms accounted for 18.87% of the overall series (*n* = 10). Two patients had multiple aneurysms (4%). Overall, 48 were saccular (90.57%), 4 were fusiform (7.55%), and 1 was a V2 segment dissecting aneurysm of VA (1.89%). The mean size of saccular aneurysms was 7.52 ± 5.34 (range: 2 to 27 mm) and most were less than 7 mm (*n* = 28, 52.83%); only one at the P2 segment of PCA was larger than 25 mm. Aneurysms were almost similarly distributed between right side (*n* = 26, 49.06%) and left side (*n* = 21, 39.62%), while six aneurysms were in the midline (11.32%). CT and MRI scans identified ten thrombotic aneurysms (18.87%). Most aneurysms were located in PCA (*n* = 18, 33.96%) and PICA (*n* = 16, 30.19%). As for PCA aneurysms, the location distributions were four in the P1 segment (7.55%), eight in the P2 segment (15.09%), one in the P3 segment (1.89%), and five in the P4 segment (9.43%). Other aneurysms were found in BA (*n* = 6, 11.32%), SCA (*n* = 4, 7.55%), AICA (*n* = 4, 7.55%), and VA (*n* = 5, 9.43%). The aneurysm characteristics are available in Table 2. 

### 3.3. Surgical Technique

As shown in Table 3, the pterional approach (*n* = 12, 24%) and midline or paramedian suboccipital approaches (*n* = 16, 32%) were the most common surgical approaches. The lateral supraorbital approach was also indispensable (*n* = 7, 14%). Four patients underwent a subtemporal approach (8%), four patients underwent a retrosigmoid approach (8%), four patients underwent a far-lateral approach (8%), and three patients underwent occipital craniotomy (6%). Classified by aneurysm location, the lateral supraorbital approach was performed in four patients with BA aneurysms (66.7%), with two undergoing the pterional approach (33.3%). The pterional and lateral supraorbital approaches were equally valuable to SCA aneurysms (*n* = 2, 50%). For PCA, P1 segment aneurysm was more suitable for pterional approach (*n* = 3, 75%) and lateral supraorbital approach (*n* = 1, 25%), while P2 segment aneurysm for pterional approach (*n* = 4, 50%) and subtemporal approach (*n* = 4, 50%), P3 segment aneurysm for subtemporal approach (*n* = 1, 100%), and P4 segment aneurysm for occipital craniotomy (*n* = 5, 100%). Retrosigmoid approach (*n* = 2, 50%) and midline or paramedian suboccipital approaches (*n* = 2, 50%) were used to treat AICA aneurysms. In 16 cases of PICA aneurysms, we performed a midline or paramedian suboccipital approach in 14 patients (87.5%) and a far-lateral approach in 2 patients (12.5%). Two patients with VA aneurysms received a retrosigmoid approach (50%), and two received a far-lateral approach (50%).

Direct clipping treated 43 (86%) of overall patients. In aneurysms that were hard to microsurgical clip directly or had the risk of sacrificing the parent artery, we performed bypass and trapping in six patients (12%). Only one fusiform and thrombotic aneurysm at the distal end of the AICA was treated by trapping and thrombectomy. To assess aneurysm remnants and patency of parent, branching, and perforating arteries, all patients (100%) underwent indocyanine green videoangiography (including FLOW 800 analysis) as an intraoperative adjunct. For eight patients (16%) with complex aneurysms, microvascular Doppler was used for real-time and dynamic evaluation of vascular hemodynamics and blood flow patterns in the aneurysm sac. The mean duration of surgery was 4.10 ± 1.82 h (range: 1.5 to 10.8 h), while six patients (12%) suffered intra-procedural aneurysm rupture. For postoperative complications, patients presented with hydrocephalus in 16% of cases (*n* = 8), rebleeding or new neurological deficit in 4% of cases (*n* = 2), and intracranial infection or cerebrospinal fluid leak in 2% of cases (*n* = 1). Among patients with hydrocephalus, five (10%) received a ventriculoperitoneal shunt and two (4%) required an external ventricular drain. Forty-eight patients received postoperative DSA or CTA. Complete aneurysm obliteration was demonstrated in 47 patients (98%). Only one patient (2%) had a residual aneurysm to preserve the perforating artery patency. There was no growth or rebleeding of the residual aneurysm during the follow-up. 

### 3.4. Outcomes and Follow-Up

On admission, 22 (44%) patients had no significant functional symptoms (mRS 0–1), and 35 (70%) patients were functionally independent (mRS 0–3). There was no significant decrease in the proportion of patients with mRS 0–3 at discharge, as 34 (68%) patients had a good clinical outcome. However, 12 patients (24%) suffered a poor clinical outcome, and 4 (8%) died. Among them, two patients, confirmed by the intraoperative adjunct without a timely angiography, died from rebleeding during hospitalization. Pneumonia, respiratory failure, and general condition deterioration led to another two patients’ deaths after surgery. The rates of good clinical outcome, poor clinical outcome, and death at discharge are presented in Table 3.

**Figure 1:**
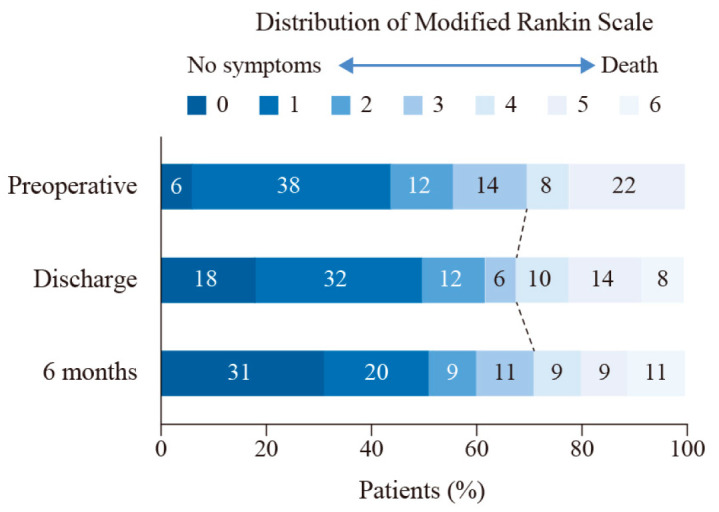
The distribution of modified Rankin Scale at admission, discharge, and six months. Shown are the percentages of patients at admission, discharge, and six months with scores from 0 to 6 on the modified Rankin scale.

The percentage of good clinical outcomes increased to 72.34% at six months, with three patients lost to follow-up. One patient died during rehabilitation due to acute coronary heart disease. Figure 1 shows the distribution of preoperative and postoperative mRS. With the mean follow-up period of 3.57 ± 1.72 years (range: 0.61 to 6.39 years), none of the patients had a recurrence of aneurysm. 

### 3.5. Illustrative Cases

#### 3.5.1. Case 1

**Figure 2:**
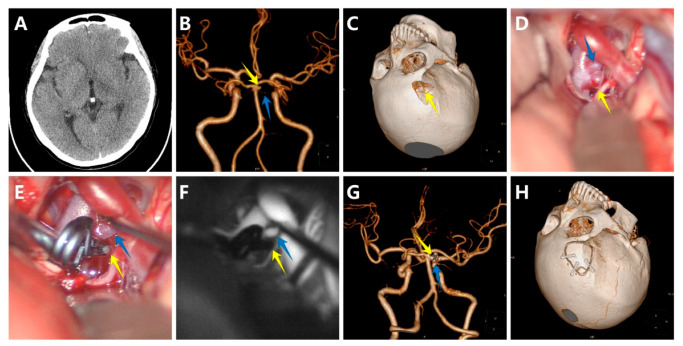
A woman with a saccular aneurysm on the bifurcation of the right superior cerebellar artery (SCA) and the basilar artery (BA). (**A**) The computed tomography scan showed no apparent abnormality; (**B**) The computed tomography angiography (CTA) revealed a 3 mm saccular aneurysm on the bifurcation of the right SCA and the BA; (**C**) Preoperative simulation of the surgical approach; (**D**) The aneurysm, SCA, and BA before clipping; (**E**) The aneurysm, SCA, and BA after clipping; (**F**) Intraoperative indocyanine green videoangiography confirmed no residual aneurysm and patency of SCA and BA; (**G**) Postoperative CTA further demonstrated complete obliteration of the aneurysm; (**H**) The small bone flap. (The yellow arrows show the aneurysm, and the blue arrows show the SCA).

A woman with no familial history, who presented with paroxysmal headache and vertigo for one month, was admitted to our hospital. A CT scan showed no apparent abnormality (Figure 2A). The CTA revealed a 3 mm saccular aneurysm on the bifurcation of the right SCA and the BA (Figure 2B). Furthermore, the aneurysm was closely associated with the origin of the SCA. Preoperative DSA showed the origin directly arising from the neck of the aneurysm. Because of the sentinel headache and to avoid sacrificing the SCA, we reached a consensus to clip the aneurysm directly. Preoperative simulation of the surgical approach suggested the neck of the aneurysm above the posterior clinoid process (Figure 2C). We finally chose the right lateral supraorbital approach. The carotid cistern was opened, and the cerebrospinal fluid was slowly released to reduce intracranial pressure, followed by sharp dissection of the liliequist membrane, right internal carotid artery, optic nerve, and BA. We quickly located the aneurysm (Figure 2D) and selected an appropriate titanium Yasargil clip to clip the aneurysm neck (Figure 2E). Intraoperative indocyanine green videoangiography and microvascular Doppler confirmed no residual aneurysm and patency of parent arteries (Figure 2F). Postoperative CTA further demonstrated complete obliteration of the aneurysm (Figure 2G) and a small bone flap (Figure 2H). The patient presented with an mRS score of 0. No aneurysm recurrence was observed after approximately three years of follow-up.

#### 3.5.2. Case 2

**Figure 3:**
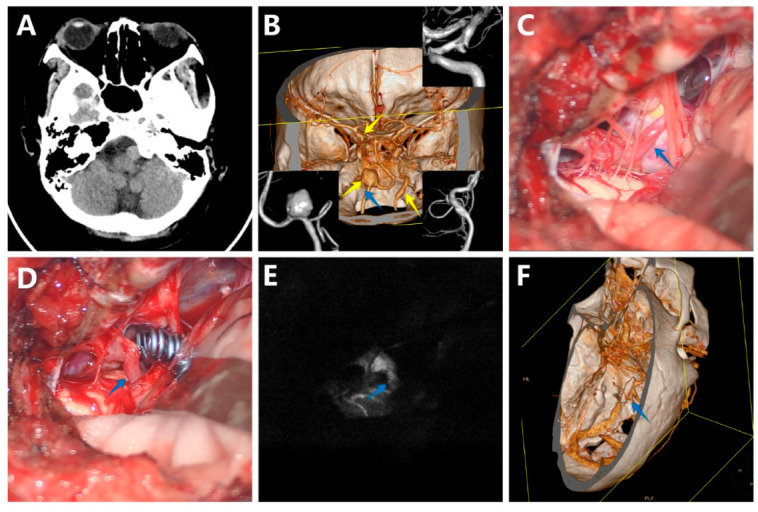
A woman with a large saccular aneurysm on the V4 segment of the right vertebral artery (VA), a small saccular aneurysm on the C7 segment of the left internal carotid artery (ICA), and a fusiform aneurysm located in the V3 segment of the left VA. (**A**) The CT scan showed a round-like mass located in the front of the medulla oblongata; (**B**) The digital subtraction angiography (DSA) and CTA disclosed a large saccular aneurysm located in the V4 segment of the right VA, a small saccular aneurysm located in the C7 segment of the left internal carotid artery, and a fusiform aneurysm located in the V3 segment of the left VA; (**C**) The aneurysm, VA, and posterior inferior cerebellar artery (PICA) before clipping; (**D**) The aneurysm, VA, and PICA after clipping; (**E**) Intraoperative indocyanine green videoangiography confirmed no residual aneurysm and patency of VA and PICA; (**F**) Postoperative CTA further demonstrated complete obliteration of the aneurysm. (The yellow arrows show the aneurysm, and the blue arrows show the PICA).

A previously healthy woman was admitted to our department because of a sudden onset of headache. The CT scan of the head did not find obvious SAH at that time, but revealed a round-like mass located in the front of the medulla oblongata (Figure 3A). The DSA and CTA disclosed a large saccular aneurysm located in the V4 segment of the right VA, a small saccular aneurysm located in the C7 segment of the left internal carotid artery projecting posteriorly and to the left, and a fusiform aneurysm located in the V3 segment of the left VA (Figure 3B). The DSA also confirmed the left PICA originated from the aneurysm neck. After carefully considering different options, local mass effect, and patient preferences, the decision was made to firstly clip the right VA aneurysm through a right far-lateral approach. With sharp arachnoid dissection, the aneurysm and lower cranial nerves were separated (Figure 3C). Titanium clips were positioned at the neck of the aneurysm (Figure 3D). Intraoperative indocyanine green videoangiography and microvascular Doppler showed patency of right VA and PICA (Figure 3E). Postoperatively, the patient suffered cerebral vasospasm, which was relieved by medical therapy with nimodipine. Early postoperative CTA confirmed complete aneurysm obliteration (Figure 3F). She was discharged with mRS 1 and without any new neurological deficit. During long-term follow-up, CTA showed no increase in the size of the two untreated aneurysms with no evidence of the aneurysm recurrence.

## 4. Discussion

Posterior circulation aneurysms comprised 3.8% to 15% of all intracranial aneurysms [2,16]. Compared to aneurysms in the anterior circulation, those in the posterior circulation were more likely to rupture [7,8,17,18]. In general, we also believed that the patients with posterior circulation aneurysms had worse clinical outcomes than those with aneurysms in other locations [9]. This could be explained by the proximity of the hypothalamus, brain stem and cranial nerves, higher tendency to be giant or fusiform aneurysms, ischemic effects, local mass effect, and dysfunction of lower cranial nerves [9,16]. Thus, treatment of posterior circulation aneurysms was particularly challenging. With advances in technology over the past several decades, the treatment tendency of intracranial aneurysms has shifted from microsurgical clipping to endovascular coiling. Endovascular coiling was considered a safe and effective treatment option with minimal invasiveness. As for aneurysms that were suitable for both neurosurgical treatment and endovascular treatment, patients allocated to endovascular coiling had a significantly lower rate of death or dependence at 1 year [8]. Further, the risk of epilepsy was greatly reduced. The endovascular treatment also showed an excellent quality-adjusted life years into the long-term follow-up [19]. Additionally, innovative endovascular techniques have continued to emerge, and the safety and efficacy of intracranial aneurysm treatment is improving [20]. Consequently, it has gradually become the preferred treatment modality of clinicians due to the shorter learning curve. 

However, the non-negligible problem was that many aneurysms only suitable for one treatment option were excluded in ISAT that only included one-fifth of the treated SAH patients [11]. This might overlook many patients who were only suitable for microsurgical clipping. Endovascular treatment of wide-neck aneurysms [21], giant thrombotic aneurysms, or aneurysms involving branching and perforating arteries was usually limited and rarely feasible. After long-term follow-up, the degree of aneurysm obliteration was significantly lower with endovascular treatment, and the dreaded recurrence and rebleeding rates were increased [12,13]. Although the usage of stents prevented coil prolapse out of aneurysms and reduced retreatment rates, thromboembolic complications and side effects associated with stenting and the antiplatelet drugs could not be ignored [22]. In addition, some of the parent arteries were severely tortuous and difficult to access [23], limiting endovascular treatment. Microsurgical occlusion was not only a viable option for some patients, but sometimes a better one. In addition to the previously mentioned higher degree of aneurysm obliteration and lower risk of recurrence or rebleeding [24], it also has the benefit of the decreased risk of hydrocephalus [25] and a lower cost [26]. Although the clinical outcome of patients with posterior circulation aneurysms was better in the endovascular treatment group at one year, this difference no longer existed after one year [27]. For these reasons, microsurgical occlusion should be considered together with endovascular coiling as an alternative treatment option. In addition, posterior circulation aneurysms were relatively rare, and detailed clinical data for microsurgical occlusion were lacking, so we reviewed this group of patients admitted to our hospital over the past six years.

According to the Swiss SOS Group, posterior circulation aneurysms in the lower third were more suitable for surgical occlusion because of easier surgical exposure and revascularization for complex aneurysms [28]. In our case series, lower-third aneurysms accounted for 39.62% (*n* = 21) of cases, while upper-third aneurysms accounted for 52.83% (*n* = 28) of cases, and middle-third aneurysms accounted for 7.55% (*n* = 4) of cases. The most common locations were PICA and PCA, which was not fully consistent with epidemiological findings. This could be explained by selective surgical option.

The mortality of neurosurgical clipping for ruptured intracranial aneurysms was 7.9% in ISAT [8]. Among their participants, only 2.7% had posterior circulation aneurysms. In the group with only posterior circulation aneurysms, postoperative mortality varied from 4.2% to 9.6% [29,30,31]. Our results indicated that the mortality was 8%, falling within the above range. Our cases were admitted with higher Hunt and Hess grades and percentage of ruptured aneurysms. The risk of poor clinical outcomes and death (mRS 4–6) at discharge was 32%, dropping to 29% at six months. Williamson et al. reported that the proportion was 91% at discharge in ruptured PICA aneurysms and improved to 63% at follow-up [9]. Nanda et al. suggested that poor preoperative clinical grades of patients with basilar artery apex aneurysms were essential predictors of clinical outcomes [32]. Another retrospective investigation observed a similar result, and the size of the aneurysm was also relevant [33]. In addition, intraoperative perforator injury, brain contusion, and cranial nerve injury should be avoided to improve the prognosis of patients.

We further compared the results of microsurgical treatment in our study with the results of endovascular treatment in other completed studies. When analyzed in ISAT, the rate of good clinical outcomes was 84% in patients randomized to endovascular coiling at two months [8]. However, we reported a significantly lower rate at discharge and six months (68% and 71%). Possible reasons for this were that the patients in our study were in worse condition on admission, aneurysms were located in posterior circulation, and the patients who were only suitable for microsurgical clipping were excluded in ISAT. The Barrow Ruptured Aneurysm Trial (BRAT) found 64.5% of patients with posterior circulation aneurysms undergoing interventional coiling had mRS > 2 at discharge [13]. In contrast, the percentage was better at 38% in our study.

The most prevalent treatment modality for posterior circulation aneurysms was direct clipping in our series. Direct clipping might be the optimal strategy for the microsurgical treatment of aneurysms. However, some complex aneurysms could not be treated by direct microsurgical clipping, clip reconstruction, or endovascular treatment. Bypass techniques expanded treatment options for these aneurysms. Some neurosurgeons recommend bypass prior to all surgical occlusion of major intracranial arteries [23]. Our center advocated bypass for large, giant, fusiform, mural calcifications, intraluminal thrombi aneurysms, or branches originating from the wall of the aneurysm to cerebral revascularization combined with clip reconstruction or occlusion of the parent artery. We performed bypass in six patients, including three fusiform aneurysms, one dissecting aneurysm, and two large thrombotic aneurysms. The location of these aneurysms was classified as VA in one patient and PCA (one in P1 segment, three in P2 segment, and one in P4 segment) in five patients. Five patients accepted postoperative angiography, which confirmed the patency of bypass and parent arteries. One patient admitted with Hunt and Hess grade IV died from rebleeding. 

Indocyanine green videoangiography was the most common intraoperative adjunct modality in our center. FLOW 800 analysis could generate a color-coded map and calculate semiquantitative cerebral blood flow parameters based on this. Intraoperative microvascular Doppler providing real-time blood flow monitoring was commonly used in bypass graft and clip reconstruction. These intraoperative adjunct techniques helped judge aneurysm remnants and the patency of parent, branching, perforating, and bypass arteries to assist the surgeon through placing the clip in a correct position at any time. Recently, temporary balloon occlusion, which could reduce the pressure of aneurysms and lower the risk of aneurysm rupture in operation, has been reported as an alternative intraoperative adjunct technique for posterior circulation aneurysms [34].

We clipped posterior circulation aneurysms in various locations by different surgical approaches. For those aneurysms in the middle and lower thirds, we commonly used the retrosigmoid approach in AICA and VA aneurysms, far-lateral approach in PICA and VA aneurysms, and midline or paramedian suboccipital approach in AICA and VA aneurysms. We could adequately expose aneurysms in the upper third through the lateral supraorbital approach, pterional approach, subtemporal approach, and occipital craniotomy. J. Hernesniemi innovated the lateral supraorbital approach in 2005 after accumulating experience with more than 2000 operations [35]. As an alternative to the pterional approach, it has the advantages of reduced temporal muscle and skin damage, shorter operation time and hospital stay, fewer surgical complications, and better masticatory or cosmetic results [36,37,38]. The lateral supraorbital approach was equally suitable for basilar bifurcation aneurysms and had better clinical outcomes [33]. The subtemporal approach and presigmoid approach were indispensable for aneurysms below the posterior clinoid process. However, the subtemporal approach had the risk of oculomotor nerve palsy, temporal lobe and the vein of Labbé injury, as well as PCA infarction [39,40]. In our case series, the lateral supraorbital approach was performed on the basilar apex, SCA, or P1 segments of PCA aneurysms, the pterional approach on the basilar apex, SCA, or P1 and P2 segments of PCA aneurysms, while the subtemporal approach on P2 or P3 segments and occipital craniotomy on all P4 segments of PCA aneurysms. The lateral supraorbital approach has turned to a routine surgical approach in our department and our experience indicated that the lateral supraorbital approach was an excellent alternative to the pterional approach for posterior circulation aneurysms above the posterior clinoid process. 

Limitations of our study included that it was a retrospective selected review of our microsurgical experience with posterior circulation aneurysms and did not compare our endovascular experience. However, this study was a re-evaluation of microsurgical occlusion therapy in the trend of endovascular treatment. Although intraoperative nerve and vascular injury affected patient outcomes, we could not assess this risk factor due to the loss of some surgical videos. Cerebral vasospasm and pulmonary infection were common complications after aneurysm surgery. However, we could not provide complete statistics on the corresponding incidence owing to the lack of records in the electronic medical record. The surgeons would probably make slight modifications to the surgical approach based on self-preference. Nonetheless, we did not think this significantly impacted the outcome. Concerning the location of the aneurysm, the corresponding number was small, so we did not distinguish the segments of their parent vessels. This study included a small number of BA aneurysms and all of them were basilar apex aneurysms. Although our data were recorded prospectively, retrospective study designs might still lead to unpredictable bias.

## 5. Conclusions

In conclusion, direct clipping and bypass should complement each other as safe and effective treatment approaches in common or complex aneurysms. The lateral supraorbital approach is a feasible, safe, and valuable surgical approach for posterior circulation aneurysms above the posterior clinoid process. Since microsurgical occlusion should be considered alongside endovascular treatment, it is essential to assist patients in formulating the most suitable treatment plan according to their characteristics and preferences.

## Figures and Tables

**Table 1:** Demographic and clinical characteristics.

Characteristics	*n* (%)
No. of patients	50
Age, years	54.92 ± 10.92 (25–77) ^1^
No. of female	28 (56%)
Presentation	
Headache	40 (80%)
Nausea and vomiting	31 (62%)
Alteration of consciousness	22 (44%)
Hoarseness and dysphagia	1 (2%)
Limb weak	2 (4%)
Seizure	1 (2%)
Comorbidities	
Hypertension	20 (40%)
Diabetes mellitus	2 (4%)
Dyslipidemia	1 (2%)
Coronary artery disease	1 (2%)
Acute kidney injury	1 (2%)
Previous treatment of another aneurysm	1 (2%)
Hunt and Hess grade	III (I–IV) ^2^
I	12 (24%)
II	9 (18%)
III	10 (20%)
IV	9 (18%)
V	3 (6%)
Modified Fisher grade	3 (1–4) ^2^
1	12 (24%)
2	6 (12%)
3	6 (12%)
4	19 (38%)

Abbreviations: No. = number; mm = millimeter. ^1^ means ± SD (range); ^2^ median (inter-quartile range, IQR).

**Table 2:** Aneurysms characteristics.

Characteristics	*n* (%)
No. of total aneurysms	53
Ruptured	43 (81.13%)
Unruptured	10 (18.87%)
Patient with multiple aneurysms	2 (4%)
Aneurysm morphology	
Saccular	48 (90.57%)
Fusiform	4 (7.55%)
Dissecting	1 (1.89%)
Size of saccular aneurysm, mm	7.52 ± 5.34 (2–27) ^1^
<7 mm	28 (52.83%)
7–12 mm	12 (22.64%)
13–24 mm	7 (13.21%)
≥25 mm	1 (1.89%)
Aneurysm location	
Basilar artery	6 (11.32%)
Superior cerebellar artery	4 (7.55%)
Posterior cerebral artery (PCA)	
PCA-P1	4 (7.55%)
PCA-P2	8 (15.09%)
PCA-P3	1 (1.89%)
PCA-P4	5 (9.43%)
Anterior inferior cerebellar artery	4 (7.55%)
Posterior inferior cerebellar artery	16 (30.19%)
Vertebral artery	5 (9.43%)
Aneurysm side	
Right	26 (49.06%)
Left	21 (39.62%)
Midline	6 (11.32%)
Thrombotic aneurysm	10 (18.87%)

Abbreviations: No. = number; mm = millimeter. ^1^ means ± SD (range).

**Table 3:** Surgical techniques, outcomes, and Follow-up.

	*n* (%)
Surgical approach	
Pterional approach	12 (24%)
Lateral supraorbital approach	7 (14%)
Subtemporal approach	4 (8%)
Retrosigmoid approach	4 (8%)
Far-lateral approach	4 (8%)
Midline or paramedian suboccipital approach	16 (32%)
Occipital craniotomy	3 (6%)
Treatment	
Clipping	43 (86%)
Bypass and trapping	6 (12%)
Trapping and thrombectomy	1 (2%)
Intraoperative adjunct	
Indocyanine green videoangiography	50 (100%)
Microvascular Doppler	8 (16%)
Duration of surgery, hours	4.10 ± 1.82 (1.5–10.8) ^1^
Intra-procedural rupture	6 (12%)
Postoperative complications	
Rebleeding	2 (4%)
New neurological deficit	2 (4%)
Hydrocephalus	8 (16%)
Intracranial infection	1 (2%)
Cerebrospinal fluid leak	1 (2%)
Aneurysm obliteration	
Complete	47 (98%)
Residual	1 (2%)
Re-operation	
External ventricular drain	2 (4%)
Ventriculoperitoneal shunt	5 (10%)
Outcome at discharge	
Good clinical outcome (mRS 0–3)	34 (68%)
Poor clinical outcome (mRS 4–5)	12 (24%)
Death (mRS 6)	4 (8%)
Aneurysm recurrence	0 (0%)
Length of follow-up, years	3.57 ± 1.72 (0.61–6.39) ^1^

Abbreviations: mRS = modified Rankin score. ^1^ means ± SD (range).

## Data Availability

The data presented in this study are available on request from the corresponding author. The data are not publicly available due to ethical restrictions.
